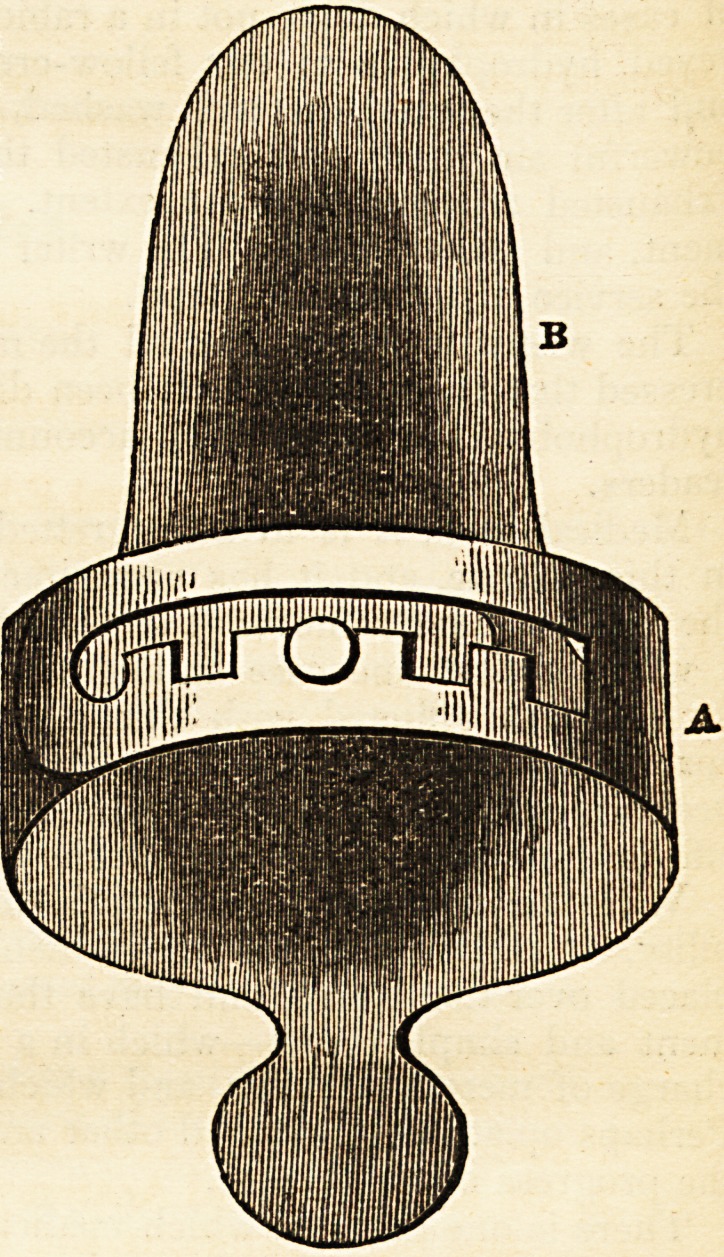# Miscellanies

**Published:** 1843-10-01

**Authors:** 


### Miscellanies.

### HYDROPHOBIA.

*To the Editor of the Northern Times.*

SIR,—Upon the return of the writer from the country at noon yesterday, he was shocked to find that a large dog, a short time before his arrival, had worried a very little dog, at his hall door, to such a degree that the steps were covered with the poor little animal's blood, which the servants immediately washed away with several buckets of water. He has since learned that a furious dog, of large size, was soon afterwards attempted to be destroyed in Monkwearmouth, but not before he had bitten, at least, three other dogs.

Every practical hint in respect to this frightful disease—hydrophobia, ought to be attended to by all classes of the community, and from actual observation, and well preserved notes, the writer will transcribe a few paragraphs for the perusal of your readers, and which, *at this moment may be of some service in these parishes.*

Some years ago, the writer was called to visit a stout young man, residing in Bishopwearmouth, on the second day of an attack of hydrophobia, who had been bitten by his own dog about four weeks previous to the attack. This young man died the day following, displaying all the distressing symptoms of the disease.

Two other persons had been bitten by the same dog. They were greatly alarmed at witnessing the progress of the disease, and placed themselves under the sole management of the writer. The bitten parts, which were in a state of ulceration, were well washed, and dressed with savin ointment. The system was kept free by the use of cooling aperients. From ten to fifteen grains of blue pill were given daily, in order that the salivary glands might be freely acted upon. The *virus* of hydrophobia is conveyed by the saliva of the rabid animal at the instant the bite is inflicted, and it was on this account that, as a prophylactic the mercury was pushed till the salivary glands became affected.

This plan was continued in these two cases for four or five weeks, and was only discontinued after the ulcers were healed up—during the use of dressings with savin ointment. These two individuals never shewed the slightest symptom of hydrophobia, and enjoyed good health for many years.

Not long after that period, the writer was called to visit a young woman residing in Monkwearmouth, in her eighteenth year, who had been recently bitten by a mad dog, while she was feeding two young pigs; both of which the rabid animal bit at the same moment. Both the pigs died, displaying all the symptoms of hydrophobia, as witnessed by the late Dr. Happer.

The writer was fortunately enabled to calm her fears, and obtain confidence in his experience. Dr. Happer, at the request of the writer, removed all the bitten surface, and took from a vein of her arm thirty ounces of blood; aperients were now administered, and the plan mentioned above was most rigorously pursued, and it is pleasing to add, that this young person continued for many years in good health.

In the year 1824, a Dalmatian, or coach dog, of great size, refused to attend to the wishes of the writer's servant, and squatted in his gite, in the stable. The servant pursued him, very foolishly, and laid hands upon him, when the animal bit him severely upon the ball of the thumb. The writer having read accounts of cases in which dogs, not in a rabid state, but under great irritation, had conveyed hydrophobia to our fellow-creatures, took his servant to the Infirmary, and after the hand was well washed, he placed it under the influence of a very powerful air-pump and exhausted the air till the blood covered the plate of the exhausted receiver to some extent. The wound was dressed with simple ointment, and soon healed. The writer saw this individual to-day, who is now in the service of a personal friend.

The writer is aware that all the members of the faculty of medicine are impressed that there has not yet been discovered that great desideratum, a cure for hydrophobia. It is on that account that these facts are placed before your readers.

Medical men, it is to be regretted, have too frequently used heroic remedies in this disease, and it has sometimes been a question whether the remedies or the disease have removed the unfortunate patients from their severe afflictions! I will mention one case amongst many—Mr. Cripps, of Liverpool, prescribed for a patient afflicted with this disease, twenty drops of croton oil in a short space of time—when it is notorious that one single drop of this medicine has been known to cause most violent symptoms, from its highly acrid and irritating effects upon the mucous membrane of the intestines.

Were the writer called to visit a case of hydrophobia, he would, in all probability, in addition to the above-mentioned remedies, order large blisters to be placed over the fauces, and have them dressed with equal parts of blue ointment and simple cerate—which in a short time would cause an abundant discharge of mercurial saliva, and which could be readily kept up by the blue pill. Perhaps opiate enemata, and other antispasmodic remedies, might be requisite in the progress of the disease.

There is one thing in which medical practitioners have heretofore been agreed —viz. that all regularly-educated medical men are entitled to attend hydrophobic patients, and witness the effects of the treatment:—Alas! we dare not say *method of cure,* as expressed in respect of all other medical diseases incident to our fellow-creatures.

In conclusion, the celebrated Dr. Fothergill, in treating upon this *opprobrium medicorum,* appends the following pertinent remarks:—“In order to remove uncertainty, if those who are applied to on these interesting emergencies would consider themselves as obliged, by the honour of their profession, and the ties of humanity, to note with all possible precision and impartiality, every incident in the progress of this disease, and whether they pursue the hints here suggested, or take up more rational ones from their own store, would communicate the result to the public, by this method the field of conjecture would be contracted, and our successors directed to new objects of investigation. The result would not be less honourable to those who engage in the research, than beneficial to mankind in general.”

The Writer offers his best wishes,

Bishopwearmouth, 12th Sept. 1843. W. R. C.

☞ We have reason to know that the writer of the above is the talented Dr. Reid Channy, of Sunderland.—*Editor.*

### DR. HOLT YATES' SPECULUM ORIS.

This useful little instrument was introduced to the profession by Dr. Holt Yates, of the Royal General Dispensary, in London, in the year 1836—since which time, he and some others have been in the habit of employing it with great advantage, in all cases in which an accurate and extensive view of the fauces was required. It deserves to be made more generally known. To the surgeon it is of essential service in the removal of tumors, or when called upon to perform any similar operation about the tonsils. By its means, not only may the jaws be distended without inconvenience to the patient, and the adjacent parts protected from injury, but the operator is enabled to see what he is about, take his own time, and to have *both his hands at liberty*. The instrument is so simple, it tells its own story; and being made of silver, the most fastidious person cannot object to its use. It consists of a graduated hoop (A) with a sliding button, so placed that it may be adjusted to the age of the patient, and other circumstances. Being gently pressed upon above and below, by the teeth, the Spatula (B) which is concave above, and slightly inclined downwards towards the extremity, depresses and protects the tongue,—and thus, an extensive and uninterrupted view of the fauces is obtained.

Any gentleman wishing to see the speculum, may do so, by applying to Mr. Simpson, surgical instrument maker, 55, Strand, or at St. Bartholomew's Hospital, where the instrument is employed.

---

### NOTICE TO MEDICAL PRACTITIONERS.

*Surgeons' Hall, Edinburgh, August* 30, 1843.

The Royal College of Surgeons of Edinburgh are desirous to make it publicly known that they lately represented to the Secretary of State for the Home Department, the injury occasioned to their fellows and licentiates, by the General Medical Order of the English Poor Law Commissioners, with respect to the appointment of Medical Officers to the Unions, Parishes, &c. under 4 and 5 William IV. c. 76, by which order all persons are excluded from these offices who have obtained their Medical qualifications in Scotland or Ireland. In consequence of this representation, the Commissioners were directed by the Home Secretary to lay a case before her Majesty's Attorney-General, for the purpose of ascertaining the state of the law under which they considered themselves bound to direct such exclusion. The Attorney-General has stated it to be his opinion, which is coincided in by Mr. Martin, another Counsel consulted, "That as far as the question of *Surgery* is concerned, those persons who have a *Surgical* Diploma, or Degree, from a Royal College or University in Scotland or Ireland, are

(in point of law) as competent to be appointed, and to act as Medical Officers under the Statute referred to, as the persons who have the diploma of the Royal College of Surgeons in London."

In consequence of this opinion, the Commissioners have intimated their intention to admit those persons who hold a Scotch or Irish Diploma or Degree in Surgery, "to the same rights under the Poor Law Amendment Act, as members of the Royal College of Surgeons of London," and "their readiness to make such modifications in their General Medical Order of the 12th March, 1842, as may be necessary for giving effect to the above recited opinion of the Attorney-General."

By order of the Royal College,

John Scott, Secretary.

---

### Royal College of Surgeons in London.

### REGULATIONS OF THE COUNCIL

Respecting the Professional Education of Candidates for the Diploma, *August* 15*th*, 1843.

I. Candidates will be required, in addition to a Certificate of being not less than twenty-one years of age, to bring proof

1. Of having been engaged in the acquirement of professional knowledge for not less than four years; during which period they must have studied Practical Pharmacy for six months, and have attended one year on the Practice of Physic, and three years on the Practice of Surgery, at a recognised Hospital or Hospitals in the United Kingdom*;—three months being allowed for a vacation in each year.

   * *By a Resolution of the Council on the 7th of November* 1839, *no Provincial Hospital will in future be recognised by this College which contains fewer than* 100 *Patients, and no Metropolitan Hospital which contains fewer than* 150 *Patients.*

2. Of having studied Anatomy and Physiology, by attendance on Lectures and Demonstrations, and by Dissections, during three Winter Sessions, of not less than six months each.

3. Of having attended at least two Courses of Lectures on the Principles and Practice of Surgery, delivered in two distinct periods or seasons, and one Course, on each of the following subjects, viz. the Practice of Physic—Chemistry—Materia Medica—and Midwifery with Practical Instruction.

II. Members and Licentiates in Surgery of any legally constituted College of Surgeons in the United Kingdom, and Graduates in Surgery of any University requiring residence to obtain Degrees, will be admitted for examination on producing their Diploma, License, or Degree, together with proofs of being twenty-one years of age, and of having been occupied at least four years in the acquirement of professional knowledge.

III. Graduates in Medicine of any legally constituted College or University requiring residence to obtain Degrees, will be admitted for examination on adducing, together with their Diploma or Degree, proof of having completed the anatomical and surgical Education required by the foregoing Regulations, either at the School of the University where they shall have graduated, or at a recognised School or Schools in the United Kingdom.

IV. Certificates will not be recognised from any Hospital unless the Surgeons thereto be members of one of the legally constituted Colleges of Surgeons in the United Kingdom; nor from any school of Anatomy, Physiology or Midwifery, unless the respective Teachers be members of some legally constituted

College of Physicians or Surgeons in the United Kingdom; nor from any School of Surgery, unless the respective Teachers be members of some legally constituted College of Surgeons in the United Kingdom.

V. Certificates will not be received on more than one branch of science from one and the same Lecturer: but Anatomy and Physiology—Demonstrations and Dissections—will be respectively considered as one branch of Science.

VI. Certificates will not be received from Candidates for the Diploma who have studied in London, unless they shall have registered their Tickets at the College, as required by the Regulations, during the last ten days of January, April and October in each year; nor from Candidates who have studied elsewhere, unless their names regularly appear in the Registers transmitted from their respective Schools.

---

N.B. In the Certificates of attendance on Hospital Practice and on Lectures, it is required that the dates of commencement and termination be clearly expressed; and no interlineation, erasure, or alteration will be allowed.

Blank forms of the required Certificates may be obtained on application to the Secretary, to whom they must be delivered, properly filled up, ten days before the Candidate can be admitted to Examination; and all such Certificates are retained at the College.

By order of the Council,
EDMUND BELFOUR, *Secretary.*

---

### PROFESSIONAL SUCCESS—GRAVITY VERSUS HILARITY.

Our contemporary, the Gazette, has favoured the profession with an Essay on Professional Success, which is good in its way, though not exactly after the manner of Dean Swift, nor Rabelais, nor Goldsmith, nor even quite so light as Addison's. But *n'importe,* it inculcates some very sound decorum and morality, and, if somewhat prosy, shares that fault in common with many excellent discourses. But the point to which we intend to allude is, one generally discussed by our contemporary—the advantages of seriousness. He observes—

"Seriousness, not occasional but habitual; earnestness, not affected but real, are useful in securing the confidence of the sick, and indispensable in the studies which prepare us for practice: and if the unvarying liveliness of a mercurial temperament, seems, in some instances, to have been a prime element of professional success, we may rely on it that such unwearied cheerfulness has only lightened the labours of conscientious activity; that it has been kept up by a consciousness of duties performed, not used as a substitute for duties neglected. Of those who can set the table in a roar, how many can leave it at the calls of duty?"

True as this may be, it cannot be deemed new, as their very charter designates the members of the College of Physicians "viri *tristes* et docti," and, no doubt, their solemnity stands them in stead. Who has not remarked some learned man, bending over a dubious stool, with a face in which are displayed all that seriousness and earnestness, so powerfully inculcated by our contemporary; and who, at the same time, has not noted the admiration which such philosophical devotedness to the cause of science and his patient, has excited in the attendant mother, or the aunt, or, above all, in the grandmother? The well weighed words, the Burleigh shake, of a professional opinion, as dark as the darkest Delphic oracle, and quite as well suited to whatever may turn up, are parts of that gravity which must pervade the sayings and doings of a doctor, and to the uninitiated, savour a little of humbug. In fact it is often, in this composite world of ours, difficult to separate the latter abused, but well used, instrument from the " conscientious earnestness" which it represents.

It must be agreed, we think, *nem. con.*, that doctors of physic should be grave, at any rate before their patients. Who, then, is to be lively? for something like fun may surely be permitted to the faculty. We fancy it must be given up to those queer fellows the surgeons—whose business of passing catheters, tying piles, and curing claps, does not necessarily require such length of visage, or intense profundity of thought. And hence, we suppose, it is, that so many of these same surgeons have been chatty, good-humoured sorts of men, not indisposed to crack either a bottle or a joke, and have not greatly suffered in the estimation of the world notwithstanding. Far be it from us to defend this. We merely chronicle the fact, and, at the present time of day, when to look wise is almost as good as to be so, and to scorn pleasantry is held by many as a certain evidence of mind, we would caution the rising generation against being misled by the lives of such persons as Cooper or Abernethy.

After all, we believe as good a precept as any, and as short, is this—*study* your profession *well*, and *practise* it *honorably*. The more you adhere to this golden rule, the less necessity there will be for affectation, hypocrisy, or humbug. A good man's own heart will tell him where a joke would be cruel—a solemn sentence cant; and the higher a man's character stands for probity and for professional ability, the less need will there be for trimming his manners to any artificial standard.

---

### Dinner to Sir Benjamin Brodie.

Our contemporary, the Gazette, contained a very accurate account of the dinner given to Sir Benjamin Brodie, at Willis's Rooms, in August last, on the occasion of presenting that gentleman with a medal. The profession is too well acquainted with the private and the public claims of Sir Benjamin Brodie on its estimation, to make it necessary for us to dwell upon that theme, however grateful. It would be hard indeed if kindliness towards his brethren of the most sterling quality—a sympathy for merit not confined, as it often is, to idle protestations and barren wishes, but extending to liberal and generous acts—a simple, unaffected, and genuine courtesy—conduct in his profession sans peur et sans reproche—a philosophical mind that not only confers success upon his practice, but makes it a study and a model—a sense of principle and duty which, much talked about yet rarely seen, is to him no effort, but a habit—and last, not least, a modesty which disclaims the merit that all except itself can see, and shuns the notoriety for which others live—it would be hard, we say, indeed, if this were not known, and, being known, rewarded by the affectionate admiration of his countrymen.

The very act that has given birth to the commemoration, stands alone in our professional history. Where do we see men in the prime of their faculties resigning a lucrative and influential appointment, on considerations of duty? Yet it was on those considerations only that Sir Benjamen Brodie retired from St. George's Hospital. He felt that such situations are held too long; that after years have cooled down zeal, and the press of private business interferes with the devotion of time and thought to public duties, the emoluments and dignities of office are retained to the exclusion of younger and more active men. Sir Benjamin Brodie felt this, or something like this, and with him to feel what is just is to do it. Whether his example be followed, or not, the act is, in itself, one worthy of imitation, and creditable alike to his heart and head.

Nor can he be said to be unrequited. Conscientious conviction has its own rewards, independently of popular suffrages or gratitude, and such recompense is, too frequently, all that it receives. But it has been Sir Benjamin Brodie's felicity to gather in his life, what is seldom reaped by the living, that harvest of golden opinions which is usually garnered up only for the dead. And a proud thing it must be to him to see envy silenced, and jealousy asleep; and in his latter days, which we trust will be many, to find himself held by the profession of his choice in esteem and veneration.

### MR. READ'S INSTRUMENTS.

We have often had the pleasure of directing attention to the apparatus of this ingenious mechanician. We have lately seen one of his contrivances which promises to be of much service. To his enema syringe, or stomach pump, he has attached a long flexible tube with a conical bulbous extremity and large lateral apertures, which may not only serve for the stomach, but is applicable to cases of impacted fæces in the colon. This may be introduced as high up the rectum as it will go. Then air or water may be thrown in, so as to distend the bowel; this will perhaps permit it to slip in farther, and so either air or fæces may be got rid of. Mr. Read has mentioned to us one or two striking cases, in which it has been a means of saving life. We would advise our medical friends to look to it.

## Figures and Tables

**Figure f1:**